# Effects of a Narrative-Based Psychoeducational Intervention to Prepare Patients for Responding to Acute Myocardial Infarction: A Randomized Clinical Trial

**DOI:** 10.1001/jamanetworkopen.2022.39208

**Published:** 2022-10-28

**Authors:** Polly W. C. Li, Doris S. F. Yu, Bryan P. Yan, C. W. Wong, Sunny C. S. Yue, Cecilia M. C. Chan

**Affiliations:** 1 School of Nursing, LKS Faculty of Medicine, The University of Hong Kong, Hong Kong; 2 Department of Medicine and Therapeutics, Faculty of Medicine, The Chinese University of Hong Kong, Hong Kong; 3 Department of Medicine and Geriatrics, Pok Oi Hospital, Hong Kong; 4 Department of Medicine and Geriatrics, United Christian Hospital, Hong Kong; 5 Department of Medicine, Queen Elizabeth Hospital, Hong Kong

## Abstract

**Importance:**

Despite decades of educational efforts, patients’ prolonged delays in seeking care for symptoms of acute myocardial infarction (AMI) remain the greatest obstacle to successful management of the condition.

**Objective:**

To compare the effects of a narrative-based psychoeducational intervention with a didactic educative approach on AMI survivors’ intention to seek care for AMI symptoms and on AMI knowledge.

**Design, Setting, and Participants:**

A multisite randomized clinical trial recruited community-dwelling patients aged 18 years or older with a history of AMI from 4 hospitals in Hong Kong from January 1, 2018, to January 22, 2021, and followed up participants for 1 year.

**Interventions:**

An 8-week narrative-based psychoeducational intervention aimed to create a vivid cognitive experience of complex decision-making and modeled desirable behavioral changes through nurse-led, interactive video sessions using model patients. The control group received 4 nurse-led sessions comprising education about AMI and care seeking delivered using a didactic approach.

**Main Outcomes and Measures:**

The primary outcome was the behavioral intention between the 2 groups, reflected by participants’ attitudes and beliefs about care seeking for AMI measured using the Acute Coronary Syndrome Response Index—Chinese version. The secondary outcome was AMI knowledge.

**Results:**

Six hundred and eight participants (mean [SD] age, 67.2 [8.3] years; 469 [77.1%] male) were randomized to either the narrative-based psychoeducation group (n = 304) or the didactic education group (n = 304). The psychoeducational intervention group reported greater positive changes than the control group in their attitudes (β = −1.053 [95% CI, −1.714 to −0.391]; *P* < .001) and beliefs (β = −0.686 [95% CI, −1.354 to −0.180]; *P* = .04) toward care seeking at the 3-month follow-up, and the difference was sustained at 12 months for both attitudes (β = −0.797 [95% CI, −1.477 to −0.117]; *P* = .02) and beliefs (β = −0.692 [95% CI, −1.309 to −0.012]; *P* = .047). There were no significant differences in AMI knowledge between the 2 study groups at the 3-month and 12-month time points.

**Conclusions and Relevance:**

The results of this randomized clinical trial found that a novel approach of narrative-based psychoeducation was effective in improving patients’ behavioral intention to seek care for AMI symptoms. Longer-term follow-up to evaluate actual care-seeking behavior and clinical outcomes in patients with AMI is warranted to determine the sustained effects of this intervention.

**Trial Registration:**

ChiCTR Identifier: ChiCTR-IIC-17010576

## Key Points

**Question:**

What are the effects of a narrative-based psychoeducational intervention compared with a didactic educational intervention on care-seeking intention in patients with a history of acute myocardial infarction (AMI)?

**Findings:**

In this randomized clinical trial of 608 survivors of AMI in Hong Kong, the narrative-based psychoeducational intervention, as shown through attitudes and beliefs about care seeking, significantly improved behavioral intention to seek care compared with the didactic educational intervention. No difference between groups was found in knowledge about AMI.

**Meaning:**

This trial found that a comprehensive, narrative-based educational approach may be used to prepare patients in responding to symptoms suggestive of AMI.

## Teaser

This randomized clinical trial examines the effects of a narrative-based psychoeducational intervention vs a didactic educational intervention on care-seeking intention in patients with a history of acute myocardial infarction.

## Introduction

Acute myocardial infarction (AMI) is associated with a substantial global disease burden and is a major cause of death wordwide.^1^ Timely revascularization is crucial to improving clinical outcomes in patients with AMI.^2,3^ In the past several decades, significant improvements have been made in reducing the door-to-treatment time,^4,5^ but there have been minimal changes in the delays that occur before patients arrive at hospitals.^6^ The prolonged time taken by patients to make a care-seeking decision remains the greatest obstacle to successful AMI management.^7^ A review of 23 studies^7^ found that the global mean delay in seeking medical care in patients with AMI was 3.4 hours. Such prolonged delay leaves patients with AMI at unnecessarily greater risks of morbidity and death.

Initiatives aimed at reducing prehospital delays for patients with AMI have mostly involved community education through media campaigns targeting the general public and brief counseling interventions targeting certain high-risk groups.^8^ Previous studies^9,10^ have found benefits of media campaigns at improving this prehospital delay time, but the effects were modest (17-24 minutes) and the results were not replicable when evaluated in a large-scale randomized clinical trial.^11^ For the brief counseling interventions, randomized clinical trials^11,12^ have reported conflicting results and largely found the interventions to be ineffective in reducing prehospital delays. Indeed, the major challenge is that patients make decisions under a complex framework, with multiple factors influencing their decisions. A sufficient understanding of the mechanism underlying the care-seeking behaviors of patients with AMI is crucial to inform the development of effective interventions. Members of our group conducted a study^13^ to elucidate the decision-making process for care seeking in patients with AMI by integrating several illness behavioral theories.^14,15,16,17^ The study found that seeking care for AMI symptoms involves a complicated perceptual-cognitive process that first requires patients to accurately label their symptoms. Such a cognitive process relies on a good understanding of disease manifestation and is associated with one’s perceived susceptibility to AMI. The process is further complicated for those who have atypical AMI manifestations. Although patients may successfully interpret symptoms, care seeking would happen only if they perceive the seriousness of AMI. Contextual factors, such as competing roles and behavioral prompts, may also affect their final decision to seek care.^13^

Based on our group’s previous findings,^13^ we developed a narrative-based psychoeducational intervention by integrating the principle of cognitive rehearsal with a narrative approach and applying various evidence-based teaching strategies, such as peer modeling, person-centered focus, and empowerment strategies.^18,19^ The goal of the intervention was to create a vivid experience for patients with AMI that would help them to go through every detail of the perceptual-cognitive processes required to make a care-seeking decision when experiencing AMI symptoms. We evaluated the effects of this intervention on educational and clinical outcomes, comparing these effects with those achieved using a didactic AMI educational intervention and reporting the educational outcomes, including participants’ behavioral intention to seek care for AMI symptoms and AMI knowledge.

## Methods

### Study Design

This was a multisite randomized clinical trial. The study was approved by the research ethics committees of participating hospitals and complied with the Declaration of Helsinki.^20^ All participants provided written informed consent. The trial protocol is provided in Supplement 1. This study followed the Consolidated Standards of Reporting Trials (CONSORT) reporting guideline for parallel-group randomized trials.^21^

### Participants

Participants were recruited from 4 regional hospitals in Hong Kong. Patients were eligible to participate in the study if they were aged 18 years or older, were living in the community, and had a history of AMI indicated in the electronic hospital record. Individuals were excluded if they had impaired communication or cognition or were actively undergoing treatment for a psychiatric disorder.

### Sample Size

The sample size was calculated based on the primary outcome of the study. Because no previous study has, to our best knowledge, examined the effects of an intensive educational intervention on patients with AMI, the sample size was determined on the basis of a large-scale randomized clinical trial examining the effects of a brief nursing educational intervention on care-seeking attitudes and beliefs related to AMI in patients with coronary artery disease.^22^ The effect sizes in terms of Cohen *d* for care-seeking attitudes and beliefs were 0.24 and 0.23, respectively. Using PASS 13 software, version 13.0.14 (NCSS LLC), we estimated that a sample size of 252 participants per study group would give the study 80% power with a 5% type I error level to detect an effect size as small as 0.23 on the primary outcome between the control and intervention groups at the postintervention time points. Allowing for a potential dropout rate of up to 20%, a total of 315 participants per group was needed.

### The Narrative-Based Psychoeducational Intervention

The intervention was based on social cognitive theory^23^ and used an eclectic approach that integrated behavioral modeling techniques to improve patients’ knowledge, skills, and self-efficacy regarding symptom recognition and care seeking. Following the Medical Research Council’s recommendation on development of complex interventions,^24^ a participatory approach was adopted by inviting patients with AMI to codesign the intervention, and it was pilot tested. Details of the intervention and its development process are described elsewhere.^25^ The narrative-based psychoeducational intervention was conducted by a registered nurse with experience in cardiac care. The intervention was group based, with 6 to 8 participants per group, and comprised 5 sessions spread over 8 weeks, including 4 weekly sessions of 90 minutes plus a booster session 4 weeks later. Each session began with a structured lesson on a topic that corresponded to the critical junctures that patients often encounter in their decision-making process, including symptom recognition, emotional response to a possible AMI, perceived barriers to and facilitators of care seeking, and the means of access to hospital service. Emphasis was placed on enhancing patients’ understanding of the symptom manifestations, disease pathophysiology, nature of disease progression, types of and rationale for treatment, appropriate care-seeking behaviors, and the significance of their prompt care-seeking behaviors in making a difference to their health outcomes. For each session, the cognitive input through education was followed by a cognitive rehearsal on the decision-making around care seeking. This involved displaying an interactive video that depicted a model patient enacting a scenario with the patient experiencing AMI symptoms and going through the perceptual-cognitive processes in decision-making. The narrative approach was used deliberately to create a vivid representation of all of the concerns and uncertainty that the patient had encountered in the process. The video was paused at critical junctures, and the nurse engaged participants to help them apply the knowledge they had gained from previous educational sessions to make appropriate judgments and decisions, brainstorming methods to resolve anticipated barriers and clarifying their misperceptions. Peer modeling was also used to enhance participants’ self-efficacy by inviting real patients to share their personal experiences through videos. Finally, an intensive scenario-based experience was used in the booster session to reinforce knowledge and decision-making skills gained in previous sessions. Participants were encouraged to apply what they had learned to resolve each situational dilemma. Emphasis was placed on grasping the skills of symptom recognition, handling the emotional responses upon symptom onset, overcoming perceived barriers, and mastering the decision-making process.

### Control Intervention

Participants in the control group received 4 weekly didactic educational sessions on AMI care seeking delivered by another nurse in a small-group format (also 6 to 8 participants per group). The topics included covered factual information about AMI and the appropriate response to possible AMI symptoms. The narrative approach and the booster session were not used in the control group.

### Outcome Measures

Outcome variables were measured at baseline (T0), 3 months after the intervention (T1), and 12 months after the intervention (T2). The primary outcome was behavioral intention to seek care for AMI symptoms, as reflected in care-seeking attitudes and beliefs upon symptom occurrence, measured using the Acute Coronary Syndrome Response Index—Chinese version (ACSRI-C). The attitudes subscale consists of 5 items (eg, “How sure are you that you could recognize the signs and symptoms of a heart attack in yourself?” and “How sure are you that you could tell the difference between the signs or symptoms of a heart attack and other medical problems?”). The beliefs subscale consists of 7 items (eg, “I would be embarrassed to go to the hospital if I thought I was having a heart attack but I wasn’t” and “If I thought I was having a heart attack, I would wait until I was very sure before going to the hospital”). Participants respond on a 4-point Likert scale. The higher subscale total scores (range for the attitudes subscale, 5-20; range for the beliefs subscale, 7-28) indicate more appropriate care-seeking attitudes and beliefs. The secondary outcome was AMI knowledge, which was measured using the knowledge subscale of the ACSRI-C. This subscale consists of 21 items (eg, symptoms such as jaw pain, nausea/vomiting, or slurred speech) to be answered dichotomously (yes/no). The correct items are summed (range, 0-21), and a higher total score represents greater knowledge. The ACSRI-C has good reliability (Cronbach α = 0.81) and convergent and construct validity.^26^

### Randomization and Blinding

Eligible participants were randomized in a 1:1 ratio with block randomization (block sizes: 8, 10, or 12). The block size and respective allocation sequence to the study groups were determined by a computer-generated sequence. Participants recruited to the study were allocated to the study groups in chronological order. Another independent research assistant, who was blinded to the study group allocation, collected the postintervention data. The Figure shows the patient flow diagram.

**Figure.**
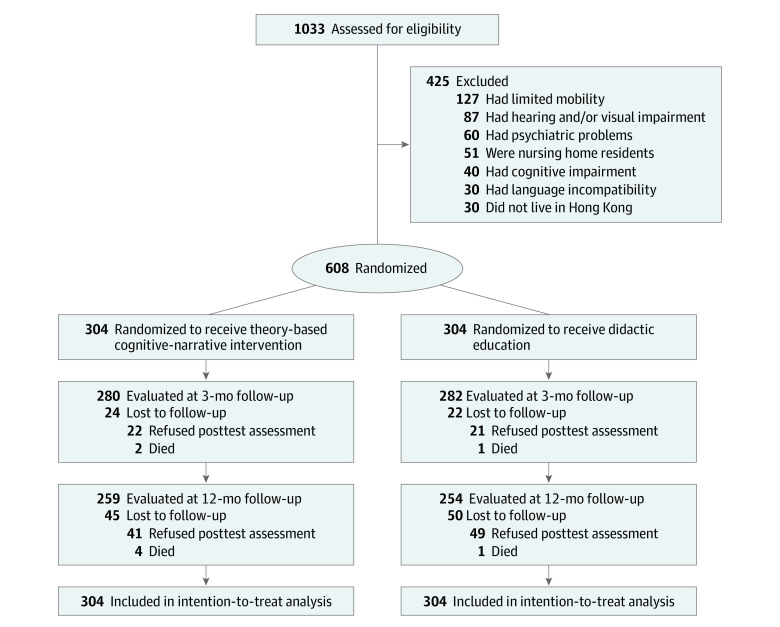
Participant Flow Diagram

### Statistical Analysis

All randomized participants were included in the intention-to-treat analysis. Baseline characteristics between the 2 study groups were compared by *t* test, χ^2^ test, or Fisher exact test as appropriate. One-way repeated-measures analysis of variance was used to analyze the within-group changes over time in care-seeking attitudes and beliefs and AMI knowledge. A generalized estimating equation model with interaction terms was used to compare the differential between-group changes of these outcomes across the time points, with adjustment for covariates. Baseline characteristics with *P* < .10 for between-group differences were considered as covariates.^27^ The mean differences in the change of scores from baseline to each time point between the 2 groups were computed, and effect sizes (Cohen *d*) were calculated. Statistical analyses were performed using SPSS software, version 27.0 (IBM Corp). All statistical tests were 2-sided, with *P* < .05 considered statistically significant.

## Results

From January 1, 2018, to January 22, 2021, a total of 1033 patients underwent assessment for eligibility. Among them, 608 patients (median [SD] age, 67.2 [8.3] years; 469 [77.1%] male and 139 [17.2%] female) were eligible and agreed to participate and being randomized to receive either the narrative-based psychoeducation (n = 304) or didactic education (n = 304) (Table 1). All respondents were of Chinese ethnicity. Most participants were married or cohabitating (505 participants [83.1%]) and retired or unemployed (458 participants [75.3%]). Approximately one-third (179 participants [29.4%]) had a primary education (6 years) or no formal education. The 2 study groups were homogeneous except that the intervention group had a higher percentage of women (81 participants [26.6%] vs 58 [19.1%]; *P* = .03) (Table 1). The numbers of participants lost to follow-up at the T1 and T2 postintervention time points were 46 (7.6%) and 95 (15.6%), respectively. There were no significant differences in demographic or clinical characteristics at baseline between those who completed the study and those who were unavailable for follow-up (eTable in Supplement 2). The overall adherence rate of the intervention group was 91.0%. Most participants in the intervention group (272 participants [89.5%]) attended at least 80% of the intervention sessions, and 7 (2.3%) missed most or all of the sessions due to medical reasons or fear of COVID-19 infection. For the control group, the overall adherence rate of the didactic education was 84.2% for all sessions, and 248 participants (81.6%) attended 3 of 4 sessions.

**Table 1.** Demographic and Clinical Characteristics of the 608 Participants at Baseline

Characteristic	Participants, No. (%)		*P* value
	Intervention group (n = 304)	Control group (n = 304)	
Age, y			
<65	97 (31.9)	120 (39.5)	.12
65-79	187 (61.5)	162 (53.3)	
≥80	20 (6.6)	22 (7.2)	
Sex			
Male	223 (73.4)	246 (80.9)	.03
Female	81 (26.6)	58 (19.1)	
Marital status			
Married or cohabitating	247 (81.3)	258 (84.9)	.12
Single, widowed, or divorced	57 (18.8)	46 (15.1)	
Living arrangement			
With family or friends	264 (86.8)	268 (88.2)	.39
Alone	40 (13.2)	36 (11.8)	
Education			
None or primary	99 (32.6)	80 (26.3)	.10
Secondary level 1 to 3	70 (23.0)	75 (24.7)	
Secondary level 4 to 7	98 (32.2)	93 (30.6)	
≥Tertiary	37 (12.2)	56 (18.4)	
Clinical history			
Percutaneous coronary intervention	227 (85.0)	217 (80.1)	.16
Coronary artery bypass graft surgery	18 (6.8)	24 (8.9)	.46
Hypertension	111 (43.2)	104 (39.2)	.41
Diabetes	93 (35.6)	90 (33.5)	.66
Hyperlipidemia	168 (57.6)	183 (69.1)	.12
Heart failure	24 (9.2)	27 (10.0)	.86
Stroke	22 (8.4)	26 (9.7)	.73
No. of previous AMIs, median (IQR)	1.01 (0.51-1.54)	1.16 (0.64-1.72)	.33
Duration since last AMI, mean (SD), mo	9.2 (2.4)	8.9 (2.7)	.12

Abbreviation: AMI, acute myocardial infarction.

### Effects of the Intervention on Attitudes and Beliefs

The analysis of variance with post hoc analysis (Table 2) indicated that the intervention group showed significant within-group improvements in attitudes (*F*_2,342_ = 126.70; *P* < .001) and beliefs (*F*_2,338_ = 39.00; *P* < .001) regarding care seeking for AMI symptoms across the postintervention time points. The control group also showed significant within-group improvements in care-seeking attitudes (*F*_2,326_ = 15.73; *P* = .04) across the time points but not their care-seeking beliefs (*F*_2,330_ = 25.27; *P* = .64). The generalized estimating equation analysis (Table 3) indicated that compared with the control group, the intervention group had a significantly greater improvement in their care-seeking attitudes for AMI from T0 to T1 (β = −1.053 [95% CI, −1.714 to −0.391]; *P* = .002). This improvement was sustained at T2 (β = −0.797 [95% CI, −1.477 to −0.117]; *P* = .02). There was also a significant between-group difference in the change in beliefs regarding care seeking from T0 to T1 (β = −0.686 [95% CI, −1.354 to −0.180]; *P* = .04), and this difference was maintained at T2 (β = −0.692 [95% CI, −1.309 to −0.012]; *P* = .047). The Cohen *d* values ranged from 0.34 to 0.39 and 0.41 to 0.58 for beliefs and attitudes, respectively, indicating small to medium effect sizes.

**Table 2.** Within-Group Changes in Outcome Variables Across the Study Time Points by 1-Way Repeated Measures Analysis of Variance^a^

Outcome	Time point	Intervention group^b^			Control group^c^		
		ACSRI-C score, mean (SD)	*F*	*P* value	ACSRI-C score, mean (SD)	*F*	*P* value
**Primary outcomes**							
Care-seeking attitudes	Baseline	10.41 (3.03)	126.70	<.001	10.99 (3.02)	15.73	.04
	3 mo	14.32 (3.18)			12.97 (3.20)		
	12 mo	13.87 (3.21)			12.60 (3.32)		
Care-seeking beliefs	Baseline	22.59 (3.13)	39.00	<.001	22.56 (3.03)	25.27	.64
	3 mo	24.98 (2.74)			23.13 (3.19)		
	12 mo	23.62 (2.79)			22.59 (3.14)		
**Secondary outcome**							
AMI knowledge	Baseline	12.22 (3.05)	31.94	<.001	12.43 (3.15)	15.87	<.001
	3 mo	13.99 (2.52)			13.58 (2.75)		
	12 mo	13.96 (2.40)			13.84 (2.24)		

Abbreviations: ACSRI-C, Acute Coronary Syndrome Response Index—Chinese version; AMI, acute myocardial infarction.

^a^ Care-seeking attitudes and beliefs and knowledge of AMI were measured with the ACSRI-C. The attitudes subscale consists of 5 items (scored from 5 to 20), and the beliefs subscale consists of 7 items (scored from 7 to 28), with higher scores indicating more appropriate care-seeking attitudes and beliefs. Knowledge about AMI was measured using the knowledge subscale, which consists of 21 items (eg, symptoms such as jaw pain, nausea/vomiting, or slurred speech) to be answered dichotomously (yes/no); the correct items are summed (range, 0-21), and a higher total score represents greater knowledge.

^b^ The intervention group received a narrative-based psychoeductional intervention comprising 5 sessions spread over 8 weeks, including 4 weekly sessions of 90 minutes plus a “booster” session 4 weeks later; there were 6 to 8 participants per group.

^c^ The control group received 4 weekly didactic educational sessions on AMI care seeking delivered by another nurse in a small-group format (6 to 8 participants per group).

**Table 3.** Between-Group Comparison of Outcome Variables Across the Study Time Points by Generalized Estimating Equation Modeling^a^

Time point	ACSRI-C score, mean (SD)		Time effect		Group effect		Interaction effect			Effect size (Cohen *d*)	
	Intervention group^b^	Control group^c^	β (95% CI)	*P* value	β (95% CI)	*P* value	β (95% CI)	*P* value		
**Primary outcomes**											
Care-seeking attitudes											
Baseline	10.41 (3.03)	10.99 (3.02)	NA		0.169 (−0.313 to 0.651)	.492					
3 mo	14.32 (3.18)	12.97 (3.20)	3.235 (0.244 to 2.756)	<.001			−1.053 (−1.714 to −0.391)	<.001		0.58	
12 mo	13.87 (3.21)	12.60 (3.32)	3.832 (3.390 to 4.274)	<.001			−0.797 (−1.477 to −0.117)	.02		0.41	
Care-seeking beliefs											
Baseline	22.59 (3.13)	22.56 (3.03)	NA		0.087 (−0.393 to 0.567)	.723					
3 mo	24.98 (2.74)	23.13 (3.19)	1.234 (0.237 to 0.770)	<.001			−0.686 (−1.354 to −0.180)	.04		0.39	
12 mo	23.62 (2.79)	22.59 (3.14)	2.559 (2.096 to 3.022)	<.001			−0.692 (−1.309 to −0.012)	.047		0.34	
**Secondary outcome**											
Knowledge of AMI										
Baseline	12.22 (3.05)	12.43 (3.15)	NA		0.096 (−0.380 to 0.807)	.962				
3 mo	13.99 (2.52)	13.58 (2.75)	1.566 (1.106 to 2.026)	<.001			−0.362 (−0.979 to 0.255)	.25	0.19	
12 mo	13.96 (2.40)	13.84 (2.24)	1.523 (1.103 to 1.942)	<.001			−0.442 (−1.074 to 0.189)	.17	0.12	

Abbreviations: ACSRI-C, Acute Coronary Syndrome Response Index—Chinese version; AMI, acute myocardial infarction; NA, not applicable.

^a^ Care-seeking attitudes and beliefs and knowledge of AMI were measured with the ACSRI-C. The attitudes subscale consists of 5 items (scored from 5 to 20), and the beliefs subscale consists of 7 items (scored from 7 to 28), with higher scores indicating more appropriate care-seeking attitudes and beliefs. Knowledge about AMI was measured using the knowledge subscale, which consists of 21 items (eg, symptoms such as jaw pain, nausea/vomiting, or slurred speech) to be answered dichotomously (yes/no); the correct items are summed (range, 0-21), and a higher total score represents greater knowledge.

^b^ The intervention group received a narrative-based psychoeductional intervention comprising 5 sessions spread over 8 weeks, including 4 weekly sessions of 90 minutes plus a “booster” session 4 weeks later; there were 6 to 8 participants per group.

^c^ The control group received 4 weekly didactic educational sessions on AMI care seeking delivered by another nurse in a small-group format (6 to 8 participants per group).

### Effect of the Intervention on AMI Knowledge

Both the intervention (*F*_2,340_ = 31.94; *P* < .001) and control (*F*_2,328_ = 15.87; *P* < .001) groups showed significant within-group improvements in AMI knowledge at postintervention time points (Table 2). However, there were no significant between-group changes in AMI knowledge at T1 (β = −0.362 [95% CI, −9.979 to 0.255]; *P* = .25) and T2 (β = −0.442 [95% CI, −1.074 to 0.189]; *P* = .17) (Table 3).

## Discussion

This randomized clinical trial found that a narrative-based psychoeducational intervention yielded greater positive changes in participants’ attitudes and beliefs about care seeking for AMI during the follow-up period compared with a didactic educative approach. Prolonged delays in making care-seeking decisions by patients remain a major obstacle to successful AMI management. This was, to our knowledge, the first study to use narrative-based psychoeducation comprising interactive education and active engagement in a virtual heart attack experience to equip AMI survivors with the skills to recognize and respond to AMI symptoms.

This result is particularly encouraging because the beliefs and attitudes regarding care seeking reflect a people’s behavioral intention, which is the most important predictor of their actual care-seeking behaviors. According to several social psychological models, such as theory of planned behavior,^28^ theory of reasoned action,^29^ and the Triandis model,^30^ intention is the key index of an individual’s mental readiness for action. Such an intention-behavior relationship is supported by the empirical findings from meta-analyses of experimental and correlational studies.^31,32^

Our results suggest greater potential for improving the behavioral intention to seek care compared with previous research, which adopted individualized brief educative and counseling interventions.^12,33,34^ The significantly improved care-seeking attitudes and beliefs found in this study may be related to integration of the cognitive rehearsal and narrative component in the intervention, which provided explicit methods to guide patients to actively express their individual concerns and beliefs throughout the decision-making process. Any misperception, hesitation, or reluctance regarding care seeking was identified and clarified. In addition, sharing by peers and positive role modeling have also been shown to be effective for improving attitudes and beliefs toward illness behaviors.^35,36^ In fact, the decision-making junctures in this intervention were informed by the cognitive and perceptual processes that members of our group identified in prior research.^13^ Factors involved in shaping the overall decision-making process in care seeking were, hence, thoroughly addressed.

The effect of the narrative-based psychoeducational intervention on behavioral intention may also be related to its compatibility with the nature of care-seeking behaviors for AMI. Other health behaviors for chronic disease management can be acquired through cognitive input, skill acquisition, practice in a clinical setting, and gradual behavioral modification,^37^ but the sudden onset of AMI implies that an alternative health education framework is needed. The narrative-based psychoeducational intervention, with the interactive video depicting a model patient enacting a scenario with the patient experiencing AMI symptoms, provided a virtual, vicarious experience for participants, enabling them to rehearse each crucial step in the decision-making process. Guided by the narrative video, participants were also exposed to different factors that shape the perceptual-cognitive process.^13^ Compared with the conventional educative approach, a narrative approach is more effective at facilitating attention, enhancing interpretation of a complex situation, rehearsing complex decision-making, recognizing possible emotional responses, and modeling desirable behaviors.^38,39^ Misperceptions or cognitive resistance can be identified for individualized counseling.^40^

Another explanation for the statistically significant effect in the primary outcome might be related to the target population of our intervention. Unlike previous studies that targeted the general public or other community-dwelling individuals,^11,34^ we targeted those patients who had a prior episode of AMI to prepare them to respond to a recurrent attack. These patients would likely perceive the intervention to be more relevant to them and, thus, be more receptive toward the intervention.

The current study found that compared with didactic education, narrative-based psychoeducation did not improve participants’ AMI knowledge. This finding could be related to the didactic AMI education for the control group, which has content and training intensity comparable to the educative counseling interventions being tested in previous studies^12,33,34^ that reported significant improvement in AMI knowledge in comparison with usual care that did not include any structured education about AMI. The significant improvement in AMI knowledge seen in our control group supports this explanation. Indeed, knowledge about the disease is a prerequisite for initiating the care-seeking process in patients with AMI, and such cognitive input is therefore needed for them to interpret symptoms accurately.

### Strengths and Limitations

This study has several strengths. First, in view of the complex challenges in the decision-making process encountered by patients with AMI, we used a participatory codesign approach to develop the intervention, which increased its relevance to patients and made it acceptable to and feasible for them. In addition, the intervention was developed on the basis of this group’s prior research informing the mechanisms underlying the decision-making process of patients with AMI.^25^ This empirical foundation may have enhanced its beneficial effects. Moreover, this was a multisite study that involved 4 major hospitals located in different districts within Hong Kong, which should increase the generalizability of these findings.

This study also has some limitations. First, the intervention comprised 5 face-to-face sessions to be implemented across 8 weeks. Such an intensive approach may limit its feasibility and application in the busy clinical setting. Future studies may test an alternative mode of delivery (eg, an online format) and a condensed version of the intervention to enhance its clinical applicability. In addition, conducting a cost-effectiveness analysis could reveal an economic benefit of the intervention. Second, female patients were underrepresented in our sample, probably because women are often older when they present with their first AMI compared with their male counterparts. This, too, may lessen the generalizability of these findings to female patients with AMI. Third, the follow-up period in our study was short and not adequately powered to detect changes in actual care-seeking delay and clinical outcomes of patients when they experience a recurrent attack, which precluded determination of the longer-term effects and clinical benefits of the intervention. Fourth, we did not have a no-treatment control group to compare the intervention effects with the usual practice in the clinical setting.

## Conclusions

Reducing care-seeking delays for patients experiencing AMI symptoms remains a challenge along the care continuum in patients with AMI. This randomized clinical trial found that a narrative-based psychoeducational intervention was effective in improving participants’ intention to seek care. Longer-term follow-up of patients’ actual care-seeking behavior and their clinical outcomes after an AMI are warranted to determine the sustained and clinical effects of this intervention.

## Notes

Supplement 1.
Trial Protocol

Supplement 2. **eTable.** Characteristics of Patients Lost to Follow-up

Supplement 3.
Data Sharing Statement
