# Supplementary material for: Effects of a Narrative-Based Psychoeducational Intervention to Prepare Patients for Responding to Acute Myocardial Infarction: A Randomized Clinical Trial
Source: JAMA Netw Open. 2022 Oct 28;5(10):e2239208. doi: 10.1001/jamanetworkopen.2022.39208 (PMC9617174; doi:10.1001/jamanetworkopen.2022.39208)
Supplement: Supplement 1. — Trial Protocol [file jamanetwopen-e2239208-s001.pdf]

Research Protocol

Study Title: The effects of a modelling-based narrative intervention on care-seeking behavioural intention and prehospital care-seeking delay in patients with acute myocardial infarction.

(a) Background of research

The importance of timely care-seeking in the context of AMI

Acute myocardial infarction (AMI) is the acute and severe manifestation of coronary artery disease (CAD), resulting from a sudden cessation of coronary blood flow due to thrombotic occlusion. It is an emergency requiring immediate medical attention. The amount of myocardial necrosis from the moment of occlusion is curvilinear with a greater extent of cell death occurring in the early hours.[17] In addition, the efficacies of reperfusion therapies are also highly time-dependent. Despite the types of reperfusion strategies, their efficacies diminish over time with a striking benefit observed within the first 2 hours of symptom onset.[17] For instance, using an analysis of 22 trials, the researchers documented a substantial reduction in mortality when the thrombolytic drugs were administered within 2 hours of symptom onset.[18] Likewise, De Luca et al. reported that the risk of 1-year mortality was raised by 7.5% for every 30-minute delay in receiving the percutaneous coronary intervention.[19] Other than the ischaemic effect, AMI patients are prone to develop fatal arrhythmias in the early hours of disease onset. According to the latest statistics,[20] majority of AMI-related deaths occur in the first hour of symptom onset are related to ischaemia-induced ventricular arrhythmias, which accounts for up to 70% of the CAD-related deaths.[20] For this reason, early access to emergency medical care for prompt defibrillation and cardiopulmonary resuscitation is of paramount importance for AMI patients. Moreover, using ambulance for transporting to hospitals enables patients to get immediate emergency care from paramedic if fatal arrhythmia occurs. In Hong Kong, up to 50% of the patients did not use ambulance during an AMI attack,[21] which greatly jeopardize their chance of survival. Indeed, AMI survivors are prone to have recurrent events and high mortality risk. A representative trial reported that up to 54% of patients presented with new myocardial ischaemia within the first year of AMI,[4] while another large population trial showed a 17.4% of 1-year risk of re-infarction.[5] Given that the time taken by patients to make their care-seeking decisions accounts for the major portion of the total ischaemic time and the high recurrent rates among the AMI survivors, there is a pressing need to develop effective interventions to promote timely care-seeking in AMI patients.

Nature of AMI care-seeking behaviour

Care-seeking is a product of how people perceive, evaluate and respond to symptoms.[22] This behavioural process not only relies on people's knowledge about the disease, but also their beliefs and attitudes regarding care-seeking.[23] Beliefs reflect people's opinions about the truth of a proposition, while attitudes are mental positions regarding something known. Both beliefs and attitudes reflect people's behavioural intention, which is the best predictor of a behaviour.[24] According to the major

theories in explaining illness behaviours, patients often make care-seeking decisions within a complex framework, where multiple factors interplay to influence their care-seeking decision. Indeed, the symptom manifestations of AMI vary considerably from time to time, as well as among different individuals. A substantial proportion of patients present with atypical symptoms,[25] which create challenges for the patients during symptom evaluation. This complexity contributes to the extensively documented AMI care-seeking delay, even in patients with a prior AMI experience.[26] Moreover, the nature of the care-seeking behaviour differs from other health-related behaviours in terms of the duration of learning history and behavioural rehearsal. For other behaviours, patients have a long learning history and plenty of opportunities for behavioural rehearsal.[27] Contrarily, care-seeking in the context of AMI involves a behaviour with little or no opportunity for learning and rehearsal,[27] which poses another challenge to patients. In such an endeavour with an absence of actual experience, providing a virtual but vicarious experience is essential for promoting timely care-seeking in AMI patients.

The complex phenomenon of AMI care-seeking behaviour

Extensive research has been published to report the factors associated with care-seeking delays in AMI patients. These factors include sociodemographic (e.g., age, gender), clinical (e.g., types of AMI, history of diabetes), cognitive (e.g., symptom attribution), emotional (e.g., fear, denial) and contextual (e.g., symptom onset time and place) factors.[28, 29] However, most of these studies focused on examining the direct effects of various factors on patients' prehospital delay.[28, 29] These studies offered important information to inform the high risk group of having a prolonged care-seeking delay, yet the underlying mechanism remains largely unknown. To address this gap in the literature, the principal investigator (PI) developed and validated an integrative model to explain the phenomenon of care-seeking delay in AMI patients.[14] This integrative model was developed on the basis of the most up-to-date empirical evidence and several behavioural theories, including Leventhal's Self-Regulatory Model of Illness Behaviour,[30, 31] the Health Belief Model[32] and Dracup's integrated model.[13] A common postulation of these theories is that upon the onset of some somatic sensations, a cognitive process is evoked to give meaning to the stimuli, which in turn shapes one's illness behaviour. According to these theories, people's care-seeking decisions can be seen as driven by two perceptual-cognitive processes, an initial 'illness labelling' process and a subsequent 'illness interpretation' process.[31, 33] At the onset of symptoms, the initial perceptual-cognitive process is initiated, where people cognitively interpret their symptoms to make a lay diagnosis. This diagnosis triggers the subsequent process of illness interpretation. Patients' interpretation and perceptions of the diagnostic label determine their tendency to seek medical care.[31, 33] This integrated theoretical model was tested by the PI in a group of 301 AMI patients.[14] The findings confirmed the two perceptual-cognitive processes in determining patients' care-seeking behaviours. According to the final model, timely care-seeking behaviours in the context of AMI depend on whether patients can correctly attribute their symptoms to AMI. This cognitive process in labelling the symptoms relies on a good understanding of the disease nature, awareness of one's susceptibility to AMI and higher alertness to atypical AMI manifestations. After this initial

labelling process, the following process in interpreting the illness in terms of perceiving severe consequences and the associated negative emotional arousal further prompt patients to seek care. In addition to these two perceptual-cognitive processes, several contextual factors also influence care-seeking behaviours. These factors include patients' perceived barriers to care-seeking and the presence of positive cues to care seeking from the significant others.[14] An effective intervention to promote timely AMI care-seeking behaviours, hence, needs to guide the patients through these perceptual-cognitive processes, assist them to identify any of the barriers and misbeliefs hindering their care-seeking behaviours throughout the process, then provide resolving methods and clarify any misunderstandings accordingly. As AMI is life-threatening and unpredictable, preparing patients to have timely care-seeking must take place before they are exposed to the situation. In such case, creating a virtual but vivid experience for an AMI attack episode is crucial to provide a platform to precisely guide the patients to rehearse every decision making in the two perceptual cognitive processes. Interactive and individualized approaches are needed to address the influence of contextual factors on actual care-seeking behaviours.

Narrative approach in health behaviours and its theoretical underpinning

A narrative form of communication is emerging as a powerful and promising alternative educational method to optimise health promotion and education.[16, 34] A narrative is defined as a "coherent story with an identifiable beginning, middle, and end that provides information about scene, characters, and conflict; raises unanswered questions or unresolved conflict; and provides resolutions."[35] Education through the narrative approach can offer a vicarious learning opportunity by creating a vivid experience for patients. A narrative approach can be achieved through a wide range of modalities, such as entertainment education using cartoon or drama, testimonials and storytelling.[16] Such an approach has the unique ability to mentally engage the audiences by transporting them into a narrative world, which leads them to engage with the events in the story. As compared to the conventional educative approach to change health behaviours, a narrative approach is more favourable in facilitating attention, enhancing interpretation of a complex situation (e.g., AMI attack), rehearsing complex decision making, recognising possibly emotional responses, modelling desirable behaviours and offering support.[34, 35] In addition, narrative communication is effective in breaking down cognitive resistance to behaviour-change messages.[36] The theoretical underpinning of the narrative approach is grounded in the observational learning of Social Cognitive Theory (SCT).[15, 35] The underlying rationale for observational learning was initially proposed by Sheffield[37] in 1961, where observation of a model in performing a certain action was proposed to provide the learner with a cognitive blueprint of the action or a standard of reference for how to perform the action, which served as a crucial guide for the learner to reproduce it later.[37] This initial idea was extended by Bandura in SCT.[38] This theory posits that modelling-based interventions play a role in influencing the outcomes through their impact on one or more of the four observational learning processes. They include: 1) the attention process which governs the sensory registration of the modelled behaviour; 2) the retention process where the modelled behaviour is symbolically coded as cognitive representations in the memory for regulating

future behaviour; 3) the production process where the component actions are integrated into patterns that require the enacting of the behaviour; and 4) motivation process where the acquired skills will be converted to overt behavioural performance.[39] The narrative approach for behavioural modelling addresses all these four processes in promoting health-related behaviours.[34, 35]

Empirical effects of narrative approach to promote health-related behaviours

Emerging evidence suggests that the narrative approach has become a powerful tool for health promotion in vulnerable populations. For instance, Houston et al. examined the effects of a narrative-based intervention delivered through an interactive DVD to promote blood pressure control.[40] The intervention included narrative stories collected from the patient population, which solicited personal experiences about hypertension control. The storytellers described how they lived with hypertension, how to interact with physicians and offered strategies to increase medication adherence. Patients with baseline uncontrolled blood pressure showed significant reduction in both systolic (11.21 mmHg) and diastolic (6.43 mmHg) blood pressures as compared to the attention placebo group.[40] Such an extent of improvement indicates that the narrative-based intervention through storytelling produced changes in blood pressure was far more effective than the other non-pharmacological behavioural interventions, with its effect even comparable to that of pharmacological interventions. Similarly, the narrative approach has been adopted to promote different types of health-behaviours. A recent meta-analysis[41] reported that narrative interventions were significant in promoting health behaviours in disease detection (e.g., mammogram test) as well as disease prevention (e.g., cancer prevention, human papillomavirus vaccination). Cole examined the effects of a series of interactive written narratives to simulate real-life situations when teaching problem-solving and decision-making skills in handling emergency situation in farm workers and coal miners.[42] The participants had to make a decision and respond at designated critical junctures. They were allowed to proceed if they made a safe option. Otherwise, they were directed to give further consideration to available alternatives and make another decision. This narrative intervention significantly improved the decision-making skills among the workers.[42] Such a narrative approach with real-life scenarios is particularly useful for training people's problem solving and decision making in situations of rare occurrence, where the chance for behavioural rehearsal is slim. The narrative approach offers an important cognitive rehearsal for their decision-making as well as the actions that they may need to implement later in their real-life situations.

The major gap in the current literature is that even though substantial evidence indicated the positive and significant effects of narrative interventions on promoting various health behaviours, no study has applied the narrative approach to promote proper care-seeking behaviours among AMI patients. Nevertheless, these previous studies offered important insight into how to develop and use a narrative approach in health promotion and educative interventions. However, these studies failed to describe the strategies to actively engage participants in the learning process. Moreover, strategies to enhance knowledge and skill retention were not adopted in their interventions, which might limit more persistent beneficial effects. Taking measures to enhance knowledge and skill retention is of particular importance when promoting prompt care-seeking behaviours in the context of AMI, as future attacks

may occur after an extended period of time.

Enhancing timely care-seeking by a narrative approach through behavioural modelling

Based on the above literature review, a narrative approach for behavioural modelling appears to have high potential to engage high risk patients to have AMI attacks in the two perceptual-cognitive processes in a guided virtual environment. In order to enhance the retention of acquired knowledge and skills, an interactive multi-media method should be integrated.[43] In particular, an interactive video presentation may be a more effective means to create virtual but vivid experience of an AMI attack to the patients. Interactive stimulating questions can be inserted throughout the video to guide multiple decision-making actions throughout the two perceptual-cognitive processes. This can allow a mental rehearsal and self-reflection, which is effective in shaping patients' future care-seeking behaviours.[44] To further strengthen and transfer behavioural modelling, extreme case model (i.e., patients who demonstrated prompt care-seeking and delay care-seeking in real life) can be invited to share their illness experience. This modelling-based approach provides a vicarious learning environment that resembles actual experience.[44] The investigation team thus hypothesises that a modelling-based narrative approach through storytelling using an interactive video presentation, interactive discussion, and extreme cases role modelling would be an effective approach to address the perplexing and persistently prolonged care-seeking delay in AMI patients.

Work done by us

This study proposes to address the findings of research conducted by the PI, which tested an integrative theoretical model explaining the care-seeking delay in AMI patients. This integrative model serves as important groundwork to guide the development of an intervention to address the issue of prolonged care-seeking delays among AMI patients. In addition, the investigation team has developed and validated a risk scoring system to estimate the risk of people developing atypical AMI symptoms.[25] Our previous work indicates that people of female gender, older age, with diabetes mellitus, a prior history of AMI and without hyperlipidaemia are more prone to present with atypical symptoms during an AMI attack. This simple scoring system will be incorporated into this study to guide the provision of tailor-made information to patients. The team of investigators, with a strong track record in cardiac care, narrative-based intervention for caregiver empowerment, patient empowerment model for self-care enhancement, and psychosocial interventions, have developed the protocol for the modelling-based narrative intervention to increase the behavioural intention for prompt care-seeking among patients at risk of developing AMI.

(b) Research plan and methodology

The aim of the study is two-fold: i) to evaluate the effects of a modelling-based narrative intervention on care-seeking beliefs, attitudes and knowledge, use of ambulance and prehospital care-seeking delays among AIM patients, and ii) to explore how and why the intervention affects the care-seeking behaviours from the patients' perspective. The behavioural intention, as reflected by the care-seeking beliefs and attitudes, are the primary outcomes, while AMI knowledge, use of ambulance and prehospital delays in care-seeking in AMI attacks are the secondary outcomes. The research hypothesis

of the first aim is that AMI patients who receive the modelling-based narrative intervention will report better changes in: i) care-seeking beliefs, ii) care-seeking attitudes, iii) AMI-related knowledge, iv) more likely to use ambulance, and v) shorter prehospital delays when AMI symptoms occur at 3, 12, and 24 months after the intervention, than those who receive didactic education on AMI care-seeking.

Study design

This is a sequential mixed-method study consisting of a randomised double-blind controlled trial and an exploratory qualitative study. The study implementation protocol is outlined in Figure 1. A research assistant (RA1) will recruit eligible participants from the cardiac clinics and wards of four regional hospitals in Hong Kong. The study sites are leading hospitals providing cardiac care services within the region. He/she will collect participants' baseline (T0) demographic data and study variables during a face-to-face interview. Block randomisation with a block size of 8, 10 or 12 will be used to ensure even distribution of participants between the two study groups. The block size and the respective allocation sequence to the intervention group receiving a modelling-based narrative intervention (by RA1) and control group receiving a didactic education (by RA2), will be determined by a computer-generated sequence. Participants chronologically recruited to the study will be allocated to the study groups by RA1 according to the computer generated sequence. Another independent RA (RA3), who is blinded to the study group allocation, will collect the post-intervention data. To detect short-term and long-term intervention effects, the outcome variables will be measured repeatedly at 3 (T1), 12 (T2) and 24 (T3) months following baseline data collection for both study groups. This extended period of outcome evaluation permits the study to detect the intervention effects on participants' actual care-seeking behaviours. Previous studies indicated that approximately 16% of these patients experienced AMI symptoms within two years.[45, 46] In the data collection telephone calls, the RA3 will enquire about whether the participants have been admitted to an emergency department with the experience of AMI symptoms. The study participants and their next of kin are strongly advised to contact the RA if they have been admitted due to a possible AMI attack.

Study participants

Patients will be eligible to join the study if they are (1) ≥18 years of age, (2) living in the community, (3) with a confirmed diagnosis of a prior AMI attack,(4) consent to participate and (5) those who can read Chinese. Individuals who do not understand Cantonese, with impaired communication ability, psychiatric problems or impaired cognitive functioning (i.e. Abbreviated Mental Test ≤6) which may hinder participation in the interventional activities will be excluded. For the RCT study, the sample size is determined on the basis of a large-scale RCT examining the effect of a brief nursing educational intervention on care-seeking knowledge, attitudes and beliefs related to AMI in people with CAD.[47] The effect sizes for knowledge, attitudes and beliefs were 0.39, 0.24 and 0.23 at 3 months after the intervention. In consideration of both clinical relevance and pervious study findings, the sample size of this study is determined to give the study adequate power to detect at least a small to medium effect size on the primary outcomes.[48] Using PASS 13 (NCSS, Kaysville, USA), it is estimated that a sample size of 252 subjects per study arm would give the study 80% power at 5%

level of significance to detect an effect size as small as 0.23 on our primary outcomes between the control and intervention arms at the post intervention time points. Further allowing for a potential dropout rate of up to 20%, a total of 315 subjects per arm will be recruited. For the qualitative phase, a criterion sampling of 30 participants who have received the modelling-based narrative intervention will be invited to join on the basis of their prehospital delay time, which will be categorised into three groups: ≤2 hours, >2 and ≤6 hours, and >6 hours. The criterion is set according to the international AMI management guidelines,[6, 49] where such cut-offs are used to categorise patients into early, intermediate and late responders. A total of 30 participants will be recruited, with 10 participants for each category. Maximum variations in the sociodemographic and clinical profile of the participants will also be adopted in the sampling procedure to illustrate a wider range of data.

Study interventions

The modelling-based narrative intervention will adopt an eclectic approach that integrate behavioural modelling and a narrative approach to promote knowledge and skill internalisation and retention (Table 1). The intervention will offer a vivid decision making experience to the participants. Participants of the intervention group will receive a modelling-based narrative intervention to be delivered by RA1. He/she will receive 3-days of intensive training from the investigation team on AMI management and care-seeking, principles and skills of group facilitation and counselling, and the ways of delivering the study protocol, accordingly.

The 7-week intervention consists of three weekly 90-minute face-to-face sessions in a small group of 6-8 participants, followed by a half-day booster session one month after the three weekly sessions (Figure 2). The small group format is adopted because it is effective in providing peer support and facilitating peer learning.[50] Each of the face-to-face sessions will begin with a brief educational presentation on a relevant topic by the intervener. These weekly topics are coherent with the critical junctures that people commonly encounter when they make care-seeking decisions in the context of AMI.[14] They include: (1) symptom recognition and risk identification, (2) emotional response to possible AMI attack and perceived barriers and facilitators to care-seeking, and (3) means of care-seeking and transportation. To optimise the effects of teaching and learning, coloured pictures and interactive skills will be used to illustrate the health information. Emphasis will be placed on enhancing patients' understanding of the symptom manifestations, disease pathophysiology, nature of disease progression, principle of treatments, appropriate care-seeking behaviours and realising the significance of their prompt care-seeking behaviours in making a difference to their health outcomes. The education content of each session complies with the latest international practice guidelines and recommendations published by the European Society of Cardiology and American Heart Association.[6, 49] The content has been validated by a panel of experts, including cardiologists, advanced practice nurses and nursing academicians in cardiac care.

After the structured educational sessions, the participants will then be offered a virtual AMI attack experience through an interactive video presentation, to consolidate their knowledge gained in previous structured educational sessions through a vivid experience. Video-based intervention is

selected as a meta-analysis reported that audio and video-based narrative interventions were more effective in changing the attitudes, intentions and behaviours when compared to paper-based narratives.[41] To engage the participants in an active learning process, a narrative storytelling approach will be adopted in each face-to-face session. A video captures a model patient in enacting the scenes of going through the perceptual-cognitive processes, including the symptom experience, symptom interpretation and the care-seeking process, to offer a mental rehearsal for the participants. The video will be paused at the four critical junctures to allow participants to outline their own decision-making process. Each pause will occur at each weekly session, with the focus on one critical moment as mentioned above. During the pause, the intervener will encourage the participants to discuss their feelings, concerns and difficulties in making their care-seeking decisions. The intervener will act as the facilitator to assist participants to solicit the application of their knowledge gained from previous educational presentation sessions, and facilitate them in making appropriate judgements and decisions. He/she has to use non-judgmental, accepting, empathetic and respectful attitudes to encourage the participants to disclose their real concerns. The intervener will also guide the participants to brainstorm resolving methods for each of the barriers that have been identified during the session. He/she will also clarify all the misbeliefs related to AMI and care-seeking. Interactive teaching strategies, such as role-play and scenario-based group activities will be adopted to facilitate the learning process. Emphasis will be placed on assisting participants in grasping the skills of symptom recognition, handling the emotional responses upon symptom onset, overcoming perceived barriers and mastering the decision-making process.

At the end of each face-to-face session, a video sharing the personal experiences of real patients will be used to enhance participants' self-efficacy through peer-modelling. In particular, the sharing will include patients with varied, but similar, backgrounds as the participants, including patients with experience of coping with the critical junctures successfully and demonstrating good recovery from the disease. Patients will also be included who delayed action when experiencing AMI symptoms and thus jeopardised their health, sustaining post-AMI complications. Such peer modelling can secure participants' understanding of how their actions can make a difference to their health outcomes.[44] The storytellers will share their real-life stories, with the emphasis placed on helping the participants to identity strategies to overcome the difficulties in decision-making.

A half-day booster session will be delivered one month after the completion of the face-to-face sessions. This booster session serves to reinforce the knowledge and decision-making skills gained from the face-to-face sessions. A scenario-based approach will be adopted to optimise participants' perceptual-cognitive processes in labelling and interpreting AMI symptoms. Five scenarios with various perceptual, social and contextual circumstances have been developed to offer a cognitive rehearsal for the participants to handle different situations (Table 2). The intervener will facilitate the participants going through the perceptual-cognitive processes for each scenario. Participants will be encouraged to express perceived barriers that exist during the decision-making course and their concerns. The intervener will encourage them to recall and apply the knowledge and skills that were

acquired in previous face-to-face sessions to resolve every situational dilemma.

A didactic education on AMI care-seeking will serve as the **control intervention**. It consists of one session to be delivered in a small group (6–8 participants/group) format by another RA (RA2). The factual information about AMI and the appropriate response towards possible symptoms will be delivered with a structured PowerPoint slide-set. Narrative approach and booster sessions will not be used in the control group. The intervener will answer questions from the participants when asked, according to the structured education content for the control group. The questions and topics discussed will be recorded for subsequent data interpretation. During the conversation, the RA will not use any wordings which is related to patient's privacy (e.g. patient's name, address etc.) and will only use a subject code to label all the audio records. Permission of audio recording of the conversation will be obtained in the informed consent and before the start of the conversation as well.

Fidelity monitoring of the study interventions

Multiple methods will be used to monitor the fidelity of the study interventions. First, the PI will randomly select five groups in each study arm for monitoring. The face-to-face sessions will be audio-taped after obtaining participants' consent. A performance checklist will be used for the purpose of fidelity monitoring. Two research fellows will review the tapes and complete the checklist after an orientation session with the PI. Secondly, a standardised manual will be developed to guide the intervention. Thirdly, the research assistants will keep reflective notes after each session and discuss the intervention with the investigation team on a regular monthly basis. The attendance of the participants will be recorded. All data will be used for data interpretation.

Outcome measures

The Acute Coronary Syndrome Response Index (ACSRI-C, Chinese version) will be used to measure the knowledge of AMI symptoms, care-seeking beliefs and attitudes upon symptoms occurrence.[51, 52] The knowledge subscale consists of 21 items to be responded dichotomously (yes/no). A four-point Likert scale is used to respond to the attitudes (5 items) and beliefs subscales (7 items). Higher scores indicate better knowledge, more appropriate care-seeking attitudes and beliefs. The ACSRI-C has good reliability (Cronbach's alpha = .81), convergent and construct validity.[51]

The use of ambulance is defined as the primary means of selecting an ambulance as the transportation modality to hospitals. It will be retrieved from medical records and doubly confirmed with the participants.

The prehospital delay time is defined as the time interval between onset of acute symptoms and the documented time of arrival at a hospital's emergency department. The time of symptom onset will be elicited by patients' recall. To ensure its accuracy, a validated benchmarking technique in which the time of symptom onset is placed in the context of routine events that surround symptom onset will be used.[53, 54] The hospital arrival time will be retrieved from the patient's medical record.

Data analysis

For the RCT study, data analysis will be performed on an intention-to-treat basis. Baseline characteristics between the two study arms will be compared by t-test, chi-square or Fisher's exact test

where appropriate. A generalized estimating equation (GEE) model will be used to compare the differential changes on the outcomes across the time points T0, T1, T2 and T3 between the two study arms with adjustment for potential confounding variables. Baseline characteristics with p values < 0.25 for between-group difference will be considered as potential confounding variables.[55] GEE model can account for intra-correlated repeated measures data and accommodate missing data caused by incomplete visits or dropout, provided that the data are missed at random,[56] and thus are particularly suitable for intention-to-treat analysis without the need of imputation for missing data. All statistical analyses will be performed using IBM SPSS 23.0. All statistical tests will be two-sided and a p-value < 0.05 will be considered statistically significant. For the qualitative study, the audio-taped data will be transcribed verbatim. The investigation team will ensure accuracy of the transcription by cross-checking. Content analysis will be adopted to code the qualitative data on participants' perceptions and acceptability of the modelling-based narrative intervention.[57] The analysis will also seek to understand why and how the study intervention influences patients' care-seeking behaviours. The codes units will be organised into categories and subcategories, which will be analysed for emerging themes. The trustworthiness of the qualitative analysis will be enhanced by audio-taping of the interviews, conducting an audit trail, involving two team members to code the qualitative data independently.[58]

Figure 1. Study implementation protocol

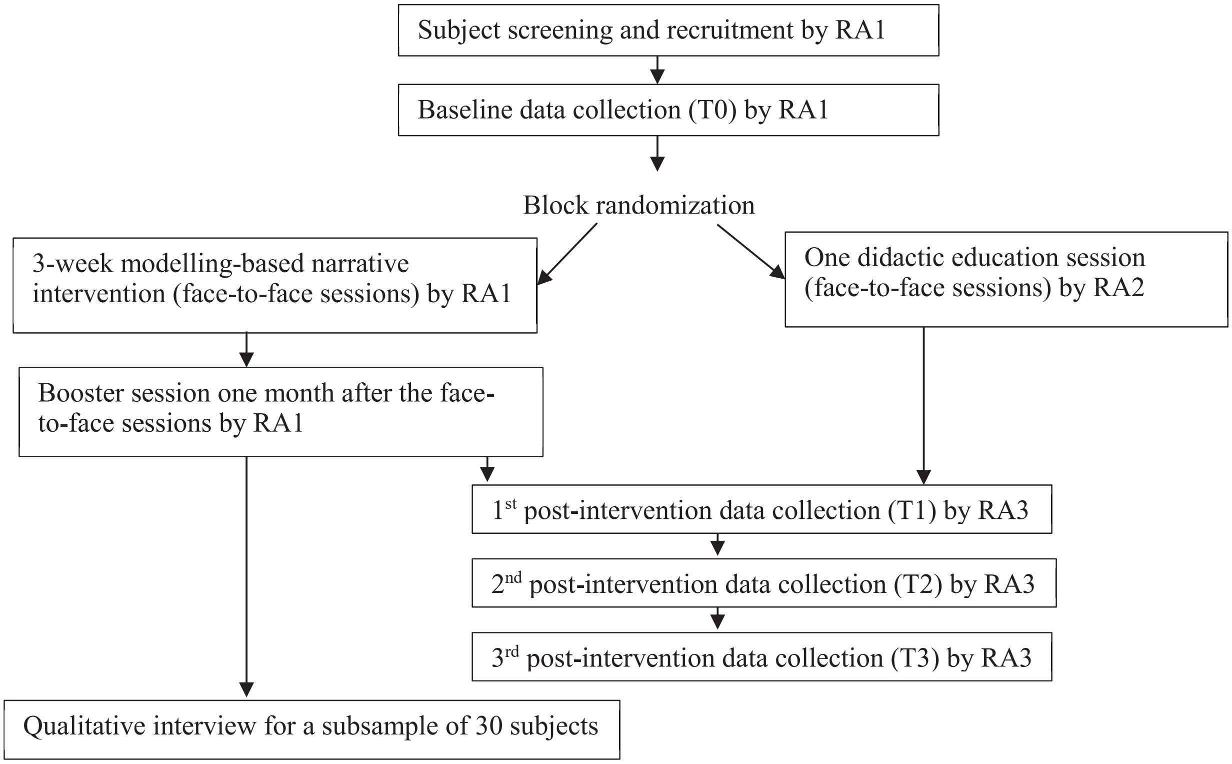

Figure 2. Graphical presentation of the modelling-based narrative intervention protocol

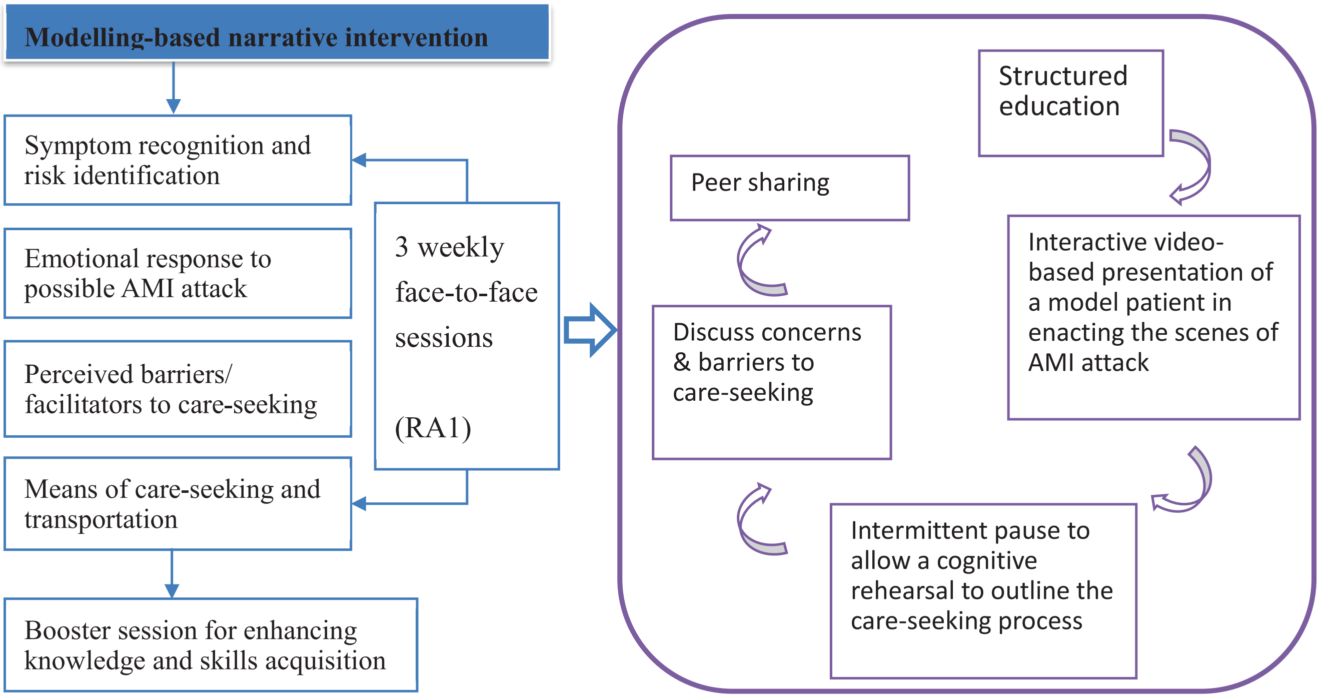

Table 1. The storyline of the narrative video-based virtual heart attack experiencing session
Background information of the model patient: **Mr. Heart**, 58 year-old man, bus driver, non-smoker, with hypertension and hyperlipidaemia. He sustained a musculoskeletal injury over his chest region a week ago

Time	Scenario
11:00pm	On a typical day, Mr. Heart has just finished a long shift of work and he is walking home from the bus terminal.
11:18pm	While he is climbing up the staircase to his house, he suddenly experiences some strange feeling of pressure over his central chest, with profuse sweating and dizziness. The pressure feeling radiates to his left shoulder and jaw → **first PAUSE for discussion**
11:20pm	Mr. Heart arrives home and immediately sits down in the living room. The first thing that comes in to his mind is that he has just had a tiring long shift and has no time for dinner. The symptoms are likely to be due to stomachache or the previous musculoskeletal injury.
11:40pm	He tried to take an antacid and a piece of bread to see if the symptoms would disappear. However, the chest discomfort persists and becomes more intense with the onset of shortness of breath after taking the medication and food. Mr. Heart starts to wonder whether the symptoms are originating from his heart. He is overwhelmingly terrified by such a possibility → **second PAUSE for discussion**
11:50pm	Mrs. Heart wakes up and notices her husband is sitting on the sofa looking very pale. After enquiring what is wrong with him, Mrs. Heart expresses her concern that the symptoms may indicate a heart problem. She suggests Mr. Heart should go to the Accident and Emergency Department for treatment
11:55pm	Mr. Heart struggles to decide whether he should go to the Accident Emergency Department immediately or wait for a little while to observe the symptoms. Many concerns relating to going or not going to the hospital flash through Mr. Heart's mind → **third PAUSE for discussion**
00:10am	Mr. Heart decides to go to the hospital as the discomfort worsens. He struggles again to decide whether he should call the ambulance or take a taxi to the hospital → **fourth PAUSE for discussion**
00:15am	Mrs. Heart activates the emergency medical service by calling 999.
00:25am	The ambulance arrives and Mr. Heart is transferred into the ambulance. When paramedic attempts to take the vital signs of Mr. Heart, he suddenly becomes unconscious. The paramedic immediately initiates resuscitation and defibrillation for Mr. Heart.
00:30am	He regains consciousness upon arrival at the Accident and Emergency Department
00:35am	Mr. Heart is diagnosed with an acute heart attack. The doctor activates the clinical pathway of heart

	attack management.
00:50am	Mr. Heart is sent to catheterisation laboratory for an emergency percutaneous coronary intervention.
02:00am	The procedure is completed and Mr. Heart is stabilised with two stents deployed at the site of the culprit lesion of a major coronary artery. He is sent to the cardiac care unit for close monitoring.

Table 2. Scenarios for the booster session of the modelling-based narrative intervention

Scenario	Information
1	Chan Tai Foon, 67-year-old lady, presenting with chest tightness and difficulty in breathing, which wakes her up at midnight. Her husband passed away 2 years ago and she lives alone.
2	Chu Yiu Keung, 55-year-old man, a private banker with an extremely heavy workload. While he is giving a presentation of his proposal to an important client, he experiences the sudden onset of chest discomfort. The pain is so intense it causes him to be unable to continue with the presentation.
3	Lee Kam Ho, 69-year-old woman, presents with gradual onset of pain over the right side of the chest while she is heading to the kindergarten to pick up her grandson at 4pm. She has to prepare the dinner for her family every evening.
4	Tsang Chi Chiu, 37-year-old man, a policeman who exercise regularly. No family history of cardiac disease. He presents with worsening chest tightness while he was exercising that week. The discomfort disappeared after a brief period of rest. While he is playing basketball tonight, he experiences the chest tightness again. However, this time, the pain persists despite a period of rest.
5	Wong Man, 82-year-old gentleman, presents with a gradual onset of dizziness and vomiting. He has type II diabetes, hypertension and coronary artery disease with stenting to 2 coronary arteries when he had a heart attack two years ago. At that time, he presented with sudden onset of severe chest pain.

References:

1. World Health Organization. Cardiovascular diseases 2015. Available from: http://www.who.int/mediacentre/factsheets/fs317/en/.
2. Centre for Health Protection. Vital statistics 2015. Available from: http://www.chp.gov.hk/en/data/4/10/27/380.html.
3. Hospital Authority. Hospital Authority strategic service framework for coronary heart disease 2013. Available from: http://www.ha.org.hk/upload/publication_42/472.pdf.
4. Goldstein JA, Demetriou D, Grines CL, Pica M, Shoukfeh M, O'Neill WW. Multiple complex coronary plaques in patients with acute myocardial infarction. New England Journal of Medicine. 2000;343(13):915-22.
5. Milonas C, Jernberg T, Lindbäck J, Agewall S, Wallentin L, Stenestrand U. Effect of angiotensin-converting enzyme inhibition on one-year mortality and frequency of repeat acute myocardial infarction in patients with acute myocardial infarction. The American Journal of Cardiology. 2010;105(9):1229-34.
6. O'Gara PT, Kushner FG, Ascheim DD, Casey DE, Chung MK, de Lemos JA, Ettinger SM, Fang JC, Fesmire FM, Franklin, BA. 2013 ACCF/AHA guideline for the management of ST-elevation myocardial infarction. Journal of the American College of Cardiology. 2013;61(4):e78-e140.
7. Denktas AE, Anderson HV, McCarthy J, Smalling RW. Total ischemic time: The correct focus of attention for optimal ST-segment elevation myocardial infarction care. JACC: Cardiovascular Interventions. 2011;4(6):599-604.
8. White HD, Chew DP. Acute myocardial infarction. The Lancet. 2008;372(9638):570-84.
9. Bagai A, Dangas GD, Stone GW, Granger CB. Reperfusion strategies in acute coronary syndromes. Circulation Research. 2014;114(12):1918-28.
10. Dracup K, McKinley S, Doering LV, Riegel B, Meischke H, Moser DK, Pelter M, Carlson B, Aitken L, Marshall A. Acute coronary syndrome: what do patients know? Archives of Internal Medicine. 2008;168(10):1049-54.
11. Goldberg RJ, Spencer FA, Fox KA, Brieger D, Steg PG, Gurfinkel E, Dedrick R, Gore JM. Prehospital delay in patients with acute coronary syndromes. American Journal of Cardiology. 2009;103(5):598-603.
12. Mooney M, McKee G, Fealy G, O'Brien F, O'Donnell S, Moser D. A review of interventions aimed at reducing pre-hospital delay time in acute coronary syndrome: what has worked and why? European Journal of Cardiovascular Nursing. 2012;11(4):445-53.
13. Dracup K, Moser DK, Eisenberg M, Meischke H, Alonzo AA, Braslow A. Causes of delay in seeking treatment for heart attack symptoms. Social Science & Medicine. 1995;40(3):379-92.

14. Li WCP, Yu SFD. Testing a model to reveal the predictive mechanism of care-seeking decisions among patients with acute myocardial infarction. Journal of Cardiovascular Nursing. 2016. [Publish Ahead of Print]
15. Bandura A. Self-efficacy: toward a unifying theory of behavioral change. Psychological review. 1977;84(2):191-215.
16. Schank R, Berman T. The pervasive role of stories in knowledge and action. Narrative impact: Social and cognitive foundations 2002. Mahwah, NJ: Lawrence Erlbaum Associates.
17. Gersh BJ, Stone GW, White HD, Holmes DR. Pharmacological facilitation of primary percutaneous coronary intervention for acute myocardial infarction: is the slope of the curve the shape of the future? Journal of American Medical Association. 2005;293(8):979-86.
18. Boersma E, Maas AC, Deckers JW, Simoons ML. Early thrombolytic treatment in acute myocardial infarction: reappraisal of the golden hour. The Lancet. 1996;348(9030):771-5.
19. De Luca G, Suryapranata H, Ottervanger JP, Antman EM. Time delay to treatment and mortality in primary angioplasty for acute myocardial infarction every minute of delay counts. Circulation. 2004;109(10):1223-5.
20. Mozaffarian D, Benjamin EJ, Go AS, Arnett DK, Blaha MJ, Cushman M, Das SR, de Ferranti S, Després JP, Fullerton HJ. Executive summary: heart disease and stroke statistics 2016 update. Circulation. 2016;133(4):447-54.
21. Li WCP, Yu SFD. Unveiling the predictive mechanisms of care-seeking delay in patients with acute myocardial infarction. Circulation. 2015;132(Suppl 3):A13428.
22. Mechanic D. The concept of illness behavior. Journal of Chronic Diseases. 1962;15(2):189-94.
23. Buckley T, McKinley S, Gallagher R, Dracup K, Moser D, Aitken LM. The effect of education and counselling on knowledge, attitudes and beliefs about responses to acute myocardial infarction symptoms. European Journal of Cardiovascular Nursing. 2007;6(2):105-11.
24. Montano DE, Kasprzyk D. Theory of reasoned action, theory of planned behavior, and the integrated behavioral model. In: GLANZ K, RIMER BK, Viswanath K, editors. Health behavior: Theory, research and practice 4th ed. CA: San Francisco: Jossey-Bass; 2015. 67-96.
25. Li WCP, Yu SFD. Recognition of atypical symptoms of acute myocardial infarction: development and validation of a risk scoring system. Journal of Cardiovascular Nursing. 2016. [Publish Ahead of Print].
26. Baxter SK, Allmark P. Reducing the time-lag between onset of chest pain and seeking professional medical help: a theory-based review. BMC Medical Research Methodology. 2013;13(1):15.
27. Raczynski JM, Finnegan JR, Zapka JG, Meischke H, Meshack A, Stone EJ, Bracht N, Sellers DE, Daya M, Robbins M. REACT theory-based intervention to reduce treatment-seeking delay for acute myocardial infarction. American Journal of Preventive Medicine. 1999;16(4):325-34.
28. Nguyen HL, Saczynski JS, Gore JM, Goldberg RJ. Age and sex differences in duration of prehospital delay in patients with acute myocardial infarction a systematic review. Circulation: Cardiovascular Quality and Outcomes. 2010;3(1):82-92.
29. Khraim FM, Carey MG. Predictors of pre-hospital delay among patients with acute myocardial infarction. Patient Education and Counselling. 2009;75(2):155-61.
30. Leventhal H, Nerenz D, Straus A. Self-regulation and the mechanisms for symptom appraisal. New York: Prodist; 1982; 55-86.
31. Leventhal H, Nerenz DR, Purse J. Illness representations and coping with health threats. Handbook of Psychology and Health. 1984; p. 219-52.
32. Becker MH. The health belief model and personal health behavior. Health Education Monographs. 1974;2:324-473.
33. Kirscht JP. The health belief model and illness behavior. Health Education Monographs. 1974;2(4):387-408.
34. Kreuter MW, Green MC, Cappella JN, Slater MD, Wise ME, Storey D, Clark EM, O'Keefe DJ, Erwin DO, Holmes K. Narrative communication in cancer prevention and control. Annals of Behavioral Medicine. 2007;33(3):221-35.
35. Hinyard LJ, Kreuter MW. Using narrative communication as a tool for health behavior change. Health Education & Behavior. 2006.
36. Dal Cin S, Zanna MP, Fong GT. Narrative persuasion and overcoming resistance. In: Knowles E, Linn J, editors. Resistance and persuasion. NJ: Mahwah Lawrence Erlbaum Associates; 2004. p. 175-91.
37. Sheffield FD. Theoretical considerations in the learning of complex sequential tasks from demonstration and practice. In: Lumsdaine AA, editor. Student response in programmed instruction1961. p. 13-32.
38. Bandura A. Social foundations of thought and action: A social cognitive theory. New Jersey: Prentice-

Hall; 1986.
39. Bandura A, Jeffrey RW. Role of symbolic coding and rehearsal processes in observational learning. Journal of personality and social psychology. 1973;26(1):122-30.
40. Houston TK, Allison JJ, Sussman M, Horn W, Holt CL, Trobaugh J, Salas M, Pisu M, Cuffee YL, Larkin D. Culturally appropriate storytelling to improve blood pressure: a randomized trial. Annals of Internal Medicine. 2011;154(2):77-84.
41. Shen F, Sheer VC, Li R. Impact of narratives on persuasion in health communication: a meta-analysis. Journal of Advertising. 2015;44(2):105-13.
42. Cole HP. A narrative approach to health behavior research and injury prevention In: Handbook of health behavior research IV. NY: Plenum; 1997.
43. Street RL, Gold WR, Manning TR. Health promotion and interactive technology: Theoretical applications and future directions: Routledge; 2013.
44. Abed MA, Himmel W, Vormfelde S, Koschack J. Video-assisted patient education to modify behavior: A systematic review. Patient Education and Counselling. 2014;97(1):16-22.
45. Dracup K, McKinley S, Riegel B, Moser DK, Meischke H, Doering LV, Davidson P, Baker H, Pelter M. A randomized clinical trial to reduce patient prehospital delay to treatment in acute coronary syndrome. Circulation: Cardiovascular Quality and Outcomes. 2009;2(6):524-32.
46. Mooney M, McKee G, Fealy G, O'Brien F, O'Donnell S, Moser D. A randomized controlled trial to reduce prehospital delay time in patients with acute coronary syndrome. The Journal of Emergency Medicine. 2014;46(4):495-506.
47. McKinley S, Dracup K, Moser DK, Riegel B, Doering LV, Meischke H, Aitken LM, Buckley T, Marshall A, Pelter M. The effect of a short one-on-one nursing intervention on knowledge, attitudes and beliefs related to response to acute coronary syndrome in people with coronary heart disease: A randomized controlled trial. International Journal of Nursing Studies. 2009;46(8):1037-46.
48. Cohen J. A power primer. Psychological Bulletin. 1992;112(1):155-9.
49. Steg PG, James SK, Atar D, Badano LP, Lundqvist CB, Borger MA, Di Mario C, Dickstein K, Ducrocq G,Fernandez-Aviles F. ESC Guidelines for the management of acute myocardial infarction in patients presenting with ST-segment elevation. European Heart Journal. 2012;33(20):2569-619.
50. Eldredge LKB, Markham CM, Kok G, Ruiter RA, Parcel GS. Planning health promotion programs: an intervention mapping approach. 3rd ed: John Wiley & Sons; 2011.
51. Cao X, Cao Y, Salamonson Y, DiGiacomo M, Chen Y, Chang S, Riegel B, Davidson PM. Translation and validation of the Chinese version of the Acute Coronary Syndrome Response Index (C-ACSRI). International Journal of Nursing Studies. 2012;49(10):1277-90.
52. Riegel B, McKinley S, Moser DK, Meischke H, Doering L, Dracup K. Psychometric evaluation of the acute coronary syndrome (ACS) response index. Research in Nursing & Health. 2007;30(6):584-94.
53. Lee H, Bahler R, Chung C, Alonzo A, Zeller RA. Prehospital delay with myocardial infarction: the interactive effect of clinical symptoms and race. Applied Nursing Research. 2000;13(3):125-33.
54. Moser DK, McKinley S, Dracup K, Chung ML. Gender differences in reasons patients delay in seeking treatment for acute myocardial infarction symptoms. Patient Education and Counselling. 2005;56(1):45-54.
55. Mickey RM, Greenland S. The impact of confounder selection criteria on effect estimation. American Journal of Epidemiology. 1989;129(1):125-37.
56. Singer JD, Willett JB. Applied longitudinal data analysis: Modeling change and event occurrence. New York: Oxford University Press; 2003.
57. Elo S, Kyngäs H. The qualitative content analysis process. Journal of Advanced Nursing. 2008;62(1):107-15.
58. Creswell JW, Miller DL. Determining validity in qualitative inquiry. Theory into Practice. 2000;39(3):124-30.
