# Supplementary material for: Effects of a Narrative-Based Psychoeducational Intervention to Prepare Patients for Responding to Acute Myocardial Infarction: A Randomized Clinical Trial
Source: JAMA Netw Open. 2022 Oct 28;5(10):e2239208. doi: 10.1001/jamanetworkopen.2022.39208 (PMC9617174; doi:10.1001/jamanetworkopen.2022.39208)
Supplement: Supplement 2. — eTable. Characteristics of Patients Lost to Follow-up [file jamanetwopen-e2239208-s002.pdf]

Supplemental Online Content

Li PWC, Yu DSF, Yan BP, Wong CW, Yue SCS, Chan CMC. Effects of a narrative-based psychoeducational intervention to prepare patients for responding to acute myocardial infarction: a randomized clinical trial. *JAMA Netw Open*. 2022;5(10):e2239208. doi:10.1001/jamanetworkopen.2022.39208

eTable. Characteristics of Patients Lost to Follow-up

This supplemental material has been provided by the authors to give readers additional information about their work.

eTable. Characteristics of Patients Lost to Follow-up

Characteristics	Intervention group (n = 304)			Control group (n = 304)		
	Lost to follow-up (n = 69) *f* (%)	Completed the study (n = 235) *f* (%)	p-value	Lost to follow-up (n = 72) *f* (%)	Completed the study (n = 232) *f* (%)	p-value
Age			0.13			0.54
<65 years	25 (36.2)	69 (29.4)		27 (37.5)	93 (40.1)	
65 – 79 years	37 (53.6)	150 (63.8)		42 (58.3)	121 (52.2)	

© 2022 Li PWC et al. *JAMA Network Open.*

≥80 years	7 (9.3)	16 (6.8)		3 (4.2)	18 (7.6)	
Gender			0.23			0.96
Male	54 (78.3)	168 (71.5)		58 (80.6)	188 (81.0)	
Female	15 (21.7)	67 (28.5)		14 (19.4)	44 (19.0)	
Marital status			0.51			0.74
Married/co-habitation	54 (78.3)	193 (82.1)		64 (88.9)	204 (87.9)	
Single/widowed/divorced	15 (21.7)	42 (17.9)		8 (11.1)	28 (12.1)	
Living arrangement			0.26			0.17
With family or friends	57 (82.6)	207 (88.1)		68 (94.4)	206 (88.8)	
Live alone	12 (17.4)	28 (11.9)		4 (5.6)	26 (11.2)	
Education			0.57			0.11
Nil/primary	18 (26.1)	80 (34.0)		17 (23.6)	65 (28.0)	
Secondary 1-3	17 (24.6)	54 (23.0)		22 (30.6)	52 (22.4)	
Secondary 4-7	25 (36.2)	71 (30.2)		26 (36.1)	66 (28.4)	
≥ Tertiary	9 (13.0)	26 (11.1)		9 (12.5)	49 (21.1)	

© 2022 Li PWC et al. *JAMA Network Open.*

Past history						
Percutaneous coronary intervention	57 (82.6)	170 (72.3)	0.14	57 (79.2)	172 (74.1)	0.82
Coronary artery bypass grafting	2 (2.9)	16 (6.8)	0.26	7 (9.7)	18 (7.8)	0.77
Hypertension	30 (43.5)	81 (41.8)	0.41	26 (36.1)	78 (40.2)	0.60
Diabetes Mellitus	23 (33.3)	70 (34.5)	0.87	20 (27.8)	70 (33.6)	0.23
Hyperlipidemia	60 (80.0)	108 (46.0)	0.28	50 (69.4)	143 (61.6)	0.77
Heart failure	5 (7.2)	19 (8.1)	0.69	7 (9.7)	20 (8.6)	0.92
Stroke	5 (7.2)	17 (7.2)	0.87	8 (12.5)	23 (9.9)	0.63
Number of previous AMI attacks (median, IQR)	0.77 (0.46)	0.84 (0.60)	0.41	0.74 (0.58)	0.80 (0.55)	0.39
Duration since last AMI episode (mean, SD)	3.87 (4.27)	4.86 (6.30)	0.23	3.30 (3.34)	4.26 (5.19)	0.07
ACSRI-C						
Care-seeking attitudes (mean, SD)	10.51 (2.93)	10.53 (3)	0.96	10.42 (3.19)	11.02 (3.09)	0.14
Care-seeking beliefs (mean, SD)	22.53 (3.12)	22.41 (3.11)	0.78	22.51 (2.79)	22.64 (2.91)	0.71
Knowledge of AMI (mean, SD)	12.49 (3.20)	12.23 (2.99)	0.52	12.18 (2.96)	12.56 (3.13)	0.34

Note: ACSRI-C = Acute Coronary syndrome Response Index – Chinese version; AMI = acute myocardial infarction. IQR = interquartile range; SD = standard deviation.

© 2022 Li PWC et al. *JAMA Network Open.*
