# Supplementary material for: Effects of a Narrative-Based Psychoeducational Intervention to Prepare Patients for Responding to Acute Myocardial Infarction: A Randomized Clinical Trial
Source: JAMA Netw Open. 2022 Oct 28;5(10):e2239208. doi: 10.1001/jamanetworkopen.2022.39208 (PMC9617174; doi:10.1001/jamanetworkopen.2022.39208)
Supplement: Supplement 3. — Data Sharing Statement [file jamanetwopen-e2239208-s003.pdf]

Data Sharing Statement

Li. Effects of a Narrative-Based Psychoeducational Intervention to Prepare Patients for Responding to Acute Myocardial Infarction. *JAMA Netw Open*. Published October 28, 2022. doi:10.1001/jamanetworkopen.2022.39208

Data
Data available: No
